# Left ventricular outcomes following multivessel PCI vs. infarct artery-only PCI in patients with acute STEMI: the Glasgow PRAMI CMR sub-study

**DOI:** 10.1186/1532-429X-17-S1-P104

**Published:** 2015-02-03

**Authors:** Kenneth Mangion, David Carrick, Alexander R Payne, John D McClure, Maureen Mason, Mark Petrie, Margaret McEntegart, Hany Eteiba, Keith G Oldroyd, Colin Berry

**Affiliations:** BHF Glasgow Cardiovascular Research Centre, University of Glasgow, Glasgow, UK; Golden Jubilee National Hospital, Clydebank, UK

## Background

In the Randomized Trial of Preventive Angioplasty in Myocardial Infarction (PRAMI; ISRCTN73028481), compared with infarct-related artery (IRA)-only PCI, additional immediate multivessel PCI (MV-PCI) of non-IRA lesions in patients with acute ST elevation myocardial infarction (STEMI) and multivessel coronary disease (MVD) improved long term prognosis. We studied left ventricular (LV) outcomes in a pre-specified cardiac magnetic resonance (CMR) sub-study.

## Methods

In a single centre prospective sub-study, PRAMI participants were invited to undergo CMR at 1.5 Tesla 1 week and 1 year after primary PCI. LV volumes and function were analysed using semi-automated software by a clinician blinded to treatment group assignment and clinical outcomes. The statistical analyses were performed by an independent statistician.

## Results

Of 465 randomised trial participants in 6 UK hospitals, 138 (30%) were enrolled in Glasgow. Eighty patients (17%) (mean age 60 years, 76% male) underwent CMR initially (n=41 (51%) in the multi-vessel PCI group; n=39 (49%) in the IRA-only group). 69 (86%) of these patients had a follow up CMR scan at 1 year (n=7 lost to follow-up, n=4 deceased). Mean (and SD) LVEF and volumes at 1 week post-MI and their change at 1 year from baseline were similar (Table 1).

**Table 1:** LVEF and volumes in randomised PRAMI trial participants (n=80) in Glasgow.

	Infarct-only PCI n = 39 (49%)	Multivessel PCI n=41 (51%)	p
1 week post-MI*			
LVEF, %	47.4 (10.8)	46.7 (11.7)	0.31
LVESVi, ml/m^2^	68.4 (15.6)	67.0 (16.9)	0.84
LVESVi, ml/m^2^	37.2 (6.6)	37.9 (15.8)	0.24
Change from baseline at 1 year*			
ΔLVEF, %	6.1 (8.9)	5.2 (11.4)	0.22
ΔLVEDVi, ml/m^2^	-3.8 (14.7)	0.7 (12.7)	0.27
ΔLVESVi, ml/m^2^	-6.6 (10.9)	-3.1 (10.4)	0.38

* mean (SD)

## Conclusions

The CMR sub-study participants represented the majority of all randomised participants in our hospital, which included one third of the PRAMI trial population. Random treatment group assignment in this CMR study was evenly balanced. LV function and volumes were similar at 1 week and 1 year post-intervention in survivors. The CMR sub-study suggests that the benefit of the preventive PCI strategy in PRAMI may not be mediated by any effects on LV function and remodelling.

## Funding

Golden Jubilee National Hospital; PRAMI was funded by Barts and the London Charity.

